# A set of serum markers detecting systemic inflammation in psoriatic skin, entheseal, and joint disease in the absence of C-reactive protein and its link to clinical disease manifestations

**DOI:** 10.1186/s13075-020-2111-8

**Published:** 2020-02-12

**Authors:** Maria V. Sokolova, David Simon, Kemal Nas, Mario M. Zaiss, Yubin Luo, Yi Zhao, Jürgen Rech, Georg Schett

**Affiliations:** 1 Department of Internal Medicine 3-Rheumatology and Immunology, Friedrich-Alexander University Erlangen-Nuremberg and Universitätsklinikum Erlangen, Ulmenweg 18, 91054 Erlangen, Germany; 2 Deutsches Zentrum fur Immuntherapie (DZI), Erlangen, Germany; 3 Division of Rheumatology and Immunology, Sakarya University School of Medicine, Sakarya, Turkey; 4 Department of Rheumatology and Immunology, West China Hospital, Sichuan University, Chengdu, China

**Keywords:** Psoriasis, Psoriatic arthritis, Enthesitis, Serum markers, Inflammation

## Abstract

**Background:**

C-reactive protein (CRP) is often normal in patients with psoriatic disease. Herein, we aimed to define markers of systemic inflammation in patients with monomorphic and polymorphic psoriatic skin, entheseal, and joint disease.

**Methods:**

Three-step approach: (i) selection of serum markers elevated in psoriatic arthritis compared healthy controls from a panel of 10 different markers reflecting the pathophysiology of psoriatic disease; (ii) testing of these selected markers as well as C-reactive protein (CRP) in a larger cohort of 210 individuals- 105 healthy controls and 105 patients with psoriatic disease with either monomorphic skin (S), entheseal (E) or joint (A) involvement or polymorphic disease with various combinations of skin, entheseal and joint disease (SE, SA, EA, SEA); (iii) testing whether tumor necrosis factor (TNF) and interleukin (IL)-17 inhibitor therapy normalizes these markers.

**Results:**

CRP was not elevated or was rarely elevated in the subgroups (S 0%, E 0%, A 20%, SE 7%, SA 33%, EA 27%, SEA 33%) despite active psoriatic disease. In sharp contrast, beta-defensin 2 and lipocalin-2 levels were elevated in the majority of patients with monomorphic skin (93% and 73%) and entheseal (both 53%), but not joint disease (27% and 20%). Conversely, elevations of calprotectin and IL-8 were found in the majority of patients with monomorphic joint disease (both 73%). IL-22 was elevated in all three monomorphic disease manifestations (S 60%, E 46%; A 60%). Furthermore, the vast majority of patients with polymorphic psoriatic disease (SE, SA, EA, SEA) showed widespread marker elevation. IL-17- and TNF inhibitor treatment significantly lowered all 5 markers of inflammation in PsA patients.

**Conclusions:**

Systemic inflammation is detectable in the majority of patients with psoriatic disease, even if CRP is normal. The respective marker pattern depends on the manifestation of psoriatic disease with respect to skin, entheseal, and joint involvement.

## Background

Psoriatic disease is a systemic inflammatory condition that affects the skin, the entheses, and the joints [1]. Psoriatic skin, entheseal, and joint disease share a common pathophysiology that is based on innate immune system activation as well as the generation of pathogenic T cells that trigger inflammation. The immune pathology in psoriasis and psoriatic arthritis (PsA) is controlled by the combination of genetic factors and extrinsic triggers, such as mechanical load, that precipitate inflammation. Inflammation in the context of psoriatic disease is considered being of systemic nature, not lastly because both conditions are associated with increased cardiovascular risk, osteoporosis, and metabolic abnormalities [1].

Despite its systemic nature, the quantification of the burden of systemic inflammation in psoriatic disease is challenging. Hence, the levels of C-reactive protein (CRP), which is commonly used to quantify systemic inflammation, are often low or absent in psoriatic disease [2]. The lack of a robust CRP signal in psoriasis and PsA is owed to the fact that interleukin (IL)-6, the essential stimulator of CRP, plays no critical role in these diseases, which is illustrated by the very limited therapeutic effect of IL-6 neutralization in psoriatic disease [3]. Lack of CRP, however, does not mean that systemic inflammation is absent but rather indicate that different markers are needed that allow better quantification of systemic inflammation in psoriasis and PsA. Notably, the respective contribution of psoriatic skin, entheseal, and joint disease to specific mediators of inflammation may be different. Thus, the production of individual mediators may be context- and tissue-dependent and therefore different whether skin, entheses, or joints are preferentially affected [4–6].

Certain mechanistic principles may help to choose the right marker panel for measuring systemic inflammatory activity in psoriasis and PsA: IL-23/IL-17 activation as well as alarmin-triggered innate immune system activation are hallmarks of the disease. Direct measurement of IL-23 and IL-17A, however, is challenging as these mediators act locally and often reach only very low concentrations in the serum [7–11]. Hence, conventional assays can detect these cytokines only in a fraction of patients and even if so, only at picomolar concentrations. In contrast, IL-17 induced proteins such as beta-defensin 2, lipocalin 2, and IL-22 reach much higher concentrations in the serum and may therefore allow more reliable assessment [12–16]. Also, molecules associated with innate immune system activation, such as the alarmins calprotectin, cathelicidin/LL-37, and pentraxin 3 triggering macrophage activation [17–19] as well as IL-8 [20–23], relevant for neutrophil activation have been reported to be involved in psoriatic disease and are found in substantial concentrations in the serum suggesting that they may qualify for measuring systemic inflammation in psoriatic disease. Finally, apart from IL-23/IL-17 pathway and innate immune system activation, angiogenesis is a key feature of the psoriatic disease [24] and elevated levels of vascular endothelial growth factor have been reported in PsA [14].

Based on these considerations, we cross-sectionally and longitudinally assessed a selected pathophysiology-based marker panel in a cohort of healthy controls and well-characterized psoriatic patients, who exhibited isolated or combined organ manifestations of psoriatic disease including the skin, the entheses, and the joints.

## Methods

### Healthy controls

Healthy subjects were recruited through a field campaign. The recruitment was prospective aiming for each 10 females and 10 males in 6 different decades (21–30, 31–40, 41–50, 51–60, 61–70, and 71–80 years) [25]. A detailed history taking and clinical examination was done in all subjects by skilled rheumatologists (AH/JR) to rule out tenderness, stiffness, swelling, and bony swelling. Subjects must have been free of present or past signs of rheumatic disease and of cancer, diabetes mellitus, cardiovascular disease (angina, myocardial infarction, stroke) as well as chronic renal, gastrointestinal, and hepatic disease. Subjects had to be tested negative for rheumatoid factor or anti-cyclic citrullinated protein antibodies (ACPA). The presence of psoriasis or a family history of psoriasis was also not allowed.

### Patients

We recruited each 15 patients in the following forms of mono- and poly-symptomatic psoriatic disease: (1) isolated psoriatic skin disease (skin, S) (2) isolated enthesitis in patients with personal or family history of psoriasis (entheses, E), (3) isolated arthritis in patients with personal or family history of psoriasis (arthritis, A), (4) psoriatic skin disease with enthesitis (SE), (5) psoriatic skin disease with arthritis (SA), (6) arthritis and enthesitis in patients with personal or family history of psoriasis (EA), and (7) the full spectrum with psoriatic skin disease, arthritis, and enthesitis (SEA). Psoriatic skin disease (S) was defined as active plaque psoriasis confirmed by the dermatologist. Enthesitis (E) was defined as tenderness at least one entheseal site of the SPARCC score over at least 6 weeks in patients with active psoriasis (SE, SEA) or patients with personal or family history of psoriasis (E, EA). The presence of entheseal inflammation has to be confirmed by a positive Power Doppler signal at least one entheseal site. Arthritis (A) was defined as joint swelling and pain for at least 6 weeks in patients with active psoriasis (SA, SEA) or patients with personal or family history of psoriasis (A, EA). To reduce the level of complexity psoriatic patients with an axial disease were not included in this study. For practical reasons, we accepted very minor skin abnormalities (PASI up to 1) in the subgroups without skin disease. All healthy subjects and patients gave written informed consent. The ethical committee of the University Clinic of Erlangen approved the study.

### Clinical assessments

Demographic (age, sex, body mass index, smoking status) and disease activity parameters for skin (psoriasis area severity index, PASI), entheseal disease (Spondyloarthritis Research Consortium of Canada, SPARCC), and joints disease (swollen joint count 66) were collected in all patients.

### Serum analyses

High-sensitivity CRP was measured by turbimetric assay with an Optilyte Analyzer from The Binding Site (Birmingham, UK). In addition, the following markers were analyzed by enzyme-linked immune sorbent assay: (1) calprotectin (S100A8/S100A9 heterodimers; R&D Diagnostics, cat. no. DS8900; normal range 481–6540 ng/mL; intra-assay precision 5.6 ng/mL; inter-assay precision 6.1 ng/mL); (2) IL-22 (R&D Diagnostics, no. D2200; normal range 0–53.3 pg/mL; intra-assay precision 56.5 pg/mL; inter-assay precision 63.0 pg/mL); (3) IL-8 (R&D Diagnostics, no. HS800; normal range 2.8–16.5 pg/mL; intra-assay precision 5.5 pg/mL; inter-assay precision 5.0 pg/mL; (4) lipocalin 2 (R&D Diagnostics, no. DLCN20; normal range 2.8–16.5 pg/mL; intra-assay precision 1.14 ng/mL; inter-assay precision 1.05 ng/mL); (5) beta-defensin 2 (Alpha Diagnostic, catalog no. 100–250-BD-2; no information on precision and normal range provided by manufacturer), [6] IL-17 (R&D Diagnostics, no. HS170; normal range not detectable − 0.4 pg/mL; intra-assay precision 2.03 pg/mL; inter-assay precision 2.09 pg/mL), [7] IL-23 (R&D Diagnostics, no. D2300B; normal range not detectable − 40.5 pg/mL; intra-assay precision 180 pg/mL; inter-assay precision 203 pg/mL); (8) VEGF (R&D Diagnostics, no. DVE00; normal range 62–707 pg/mL; intra-assay precision 29.2 pg/mL; inter-assay precision 32.8 pg/mL), [9] LL37 (Cusabio, no. E14948; no information on precision and normal range provided by manufacturer), (10) pentraxin 3 (R&D Diagnostics, no. DPTX30B; normal range not detectable − 1.36 ng/mL  pg/mL; intra-assay precision 2.12 ng/mL; inter-assay precision 2.05 ng/mL).

Measurements were done in a blinded way in duplicates by scientists not involved in the clinical patient assessment (MS, KN, YL) in a three-step approach: (i) first signal finding step, (ii) second validation step, and (iii) longitudinal step. In the signal finding step (which did not include routine CRP, 10 healthy controls and 10 patients with active polymorphic psoriatic arthritis (including skin, entheseal, and joint disease, SEA) were tested for all 10 parameters (see above) to find a difference between healthy controls and disease. Cut-offs for positive values were defined as the normal value plus 3 standard deviations. In the validation step, 105 controls and 105 disease samples (each 15 from the 7 aforementioned disease patterns) were tested for the 5 parameters (beta-defensin 2, lipocalin 2, IL-22, IL-8, calprotectin), which showed significant differences between controls and disease, as well as for CRP. And finally, in the longitudinal step, each 10 PsA patients (A, SA or SEA) receiving treatment with either TNF- or IL-17 inhibition were tested for the parameters before treatment and 3 months after initiation of treatment.

### Statistical analysis

Data were collected, organized, and analyzed through SPSS software for statistics (IBM SPSS 21.0, IBM corporation®, Armonk, NY, USA). With respect to demographic and disease-specific characteristics, categorical variables are presented as numbers and percentages, continuous variables are provided as mean ± standard deviation (SD), if not stated otherwise. Assumptions of normally distributed continuous variables were tested using quantile-quantile plots, Kolmogorov-Smirnov, and Shapiro-Wilk test. For comparison of the above-mentioned serum parameters between healthy controls and the respective disease groups, unpaired Student’s *t* test was applied. For comparison of baseline pre-treatment values and post-treatment values, paired Student’s *t* test was used. *P* values ≤ 0.05 were considered statistically significant.

## Results

### Characteristics of patients and controls

Totally, 105 healthy subjects and 105 patients with the psoriatic disease were analyzed in this study. Their demographic and disease-specific characteristics are summarized in Tables 1 and 2. Patients with the psoriatic disease were recruited according to their pattern of disease manifestation with monomorphic skin (S), entheseal (E), and joint (A, arthritis) or polymorphic (SE, SA, EA, SEA) disease. Each disease pattern was represented by 15 patients. Demographic characteristics were comparable among all groups and within each disease manifestations, the activity of the skin, entheseal, and joint disease was also comparable.

**Table 1:** Clinical parameters

	HC	S	E	A	SE	SA	EA	SEA
	*N* = 105	*N* = 105						
		*N* = 15	*N* = 15	*N* = 15	*N* = 15	*N* = 15	*N* = 15	*N* = 15
Age	53 ± 11	53 ± 8	56 ± 9	52 ± 10	54 ± 8	57 ± 8	56 ± 9	56 ± 8
Sex (F)	49.5%	53.3%	40.0	53.3%	46.6%	46.6%	49.5%	40.0%
BMI	28 ± 2.4	30 ± 3.2	28 ± 3.1	29 ± 2.6	28 ± 3.2	30 ± 2.7	29 ± 3.5	29 ± 2.5
PASI	–	8.8 ± 3.0	0.1 ± 0.3	0.2 ± 0.3	7.5 ± 2.9	9.2 ± 3.2	0.2 ± 0.3	8.0 ± 2.9
SPARCC	–	0 ± 0	4.2 ± 1.8	0 ± 0	2.8 ± 1.3	0 ± 0	3.5 ± 2.2	3.2 ± 1.3
SJC	–	0 ± 0	0 ± 0	6.1 ± 2.8	0 ± 0	5.6 ± 2.6	6.2 ± 2.6	6.4 ± 2.8

*HC* healthy controls; monomorphic psoriatic disease manifestations: *S* skin disease, *E* enthesitis, *A* arthritis; polymorphic psoriatic disease manifestations: *SE* skin disease + enthesitis, *SA* skin disease + arthritis, *EA* enthesitis + arthritis, *SEA* skin + enthesitis + arthritis, *BMI* body mass index, *PASI* psoriasis area severity index, *SPARCC* Spondyloarthritis Research Consortium of Canada Enthesitis Index, *SJC* swollen joint count. All values except sex (% females) indicate means ± SEM

**Table 2:** Laboratory parameters

HC		S	E	A	SE	SA	EA	SEA
*N* = 105		*N* = 105						
		*N* = 15	*N* = 15	*N* = 15	*N* = 15	*N* = 15	*N* = 15	*N* = 15
CRP	3.1 ± 0.1	3.4 ± 0.3	3.1 ± 0.3	6.4 ± 1.6^***^	3.0 ± 0.3	5.2 ± 0.9^***^	5.5 ± 1.1^***^	6.7 ± 1.4^***^
LC2	8.7 ± 0.5	86 ± 14^***^	44 ± 7^***^	33 ± 6^***^	93 ± 18^***^	71 ± 10^***^	50 ± 6^***^	76 ± 11^***^
BD2	0.4 ± 0.1	10 ± 2.8^***^	2.4 ± 0.4^***^	1.5 ± 1.4^***^	10 ± 2.7^***^	9.9 ± 2.2^***^	1.7 ± 0.4^***^	9.3 ± 3.1^***^
IL-22	8.6 ± 0.2	19 ± 2.6^***^	20 ± 2.6^***^	13 ± 2.3^***^	17.4 ± 1.7^***^	29 ± 5.5^***^	27 ± 3.8^***^	41 ± 5.6^***^
IL-8	4.4 ± 0.1	11 ± 2.2^***^	10 ± 1.3^***^	25 ± 6.9^***^	14 ± 2.1^***^	18 ± 4.1^***^	20 ± 5.0^***^	25 ± 6.6^***^
CP	1.3 ± 0.1	3.9 ± 0.5^***^	4.4 ± 0.6^***^	8.2 ± 1.0^***^	4.9 ± 0.6^***^	10 ± 1.6^***^	7.2 ± 1.3^***^	8.5 ± 1.6^***^

*HC* healthy controls; monomorphic psoriatic disease manifestations: *S* skin disease, *E* enthesitis, A: arthritis; polymorphic psoriatic disease manifestations: *SE* skin disease + enthesitis, *SA* skin disease + arthritis, *EA* enthesitis + arthritis, *SEA* skin + enthesitis + arthritis, *CRP* C-reactive protein, *LC2* lipocalin 2, *BD2* beta-defensin 2, *IL* interleukin, *CP* calprotectin and IL-8. All values indicate means ± SEM. Asterisks indicate significances (*p* < 0.01) compared to healthy controls

### Selection of serum markers of inflammation elevated in psoriatic arthritis

**Fig. 1:**
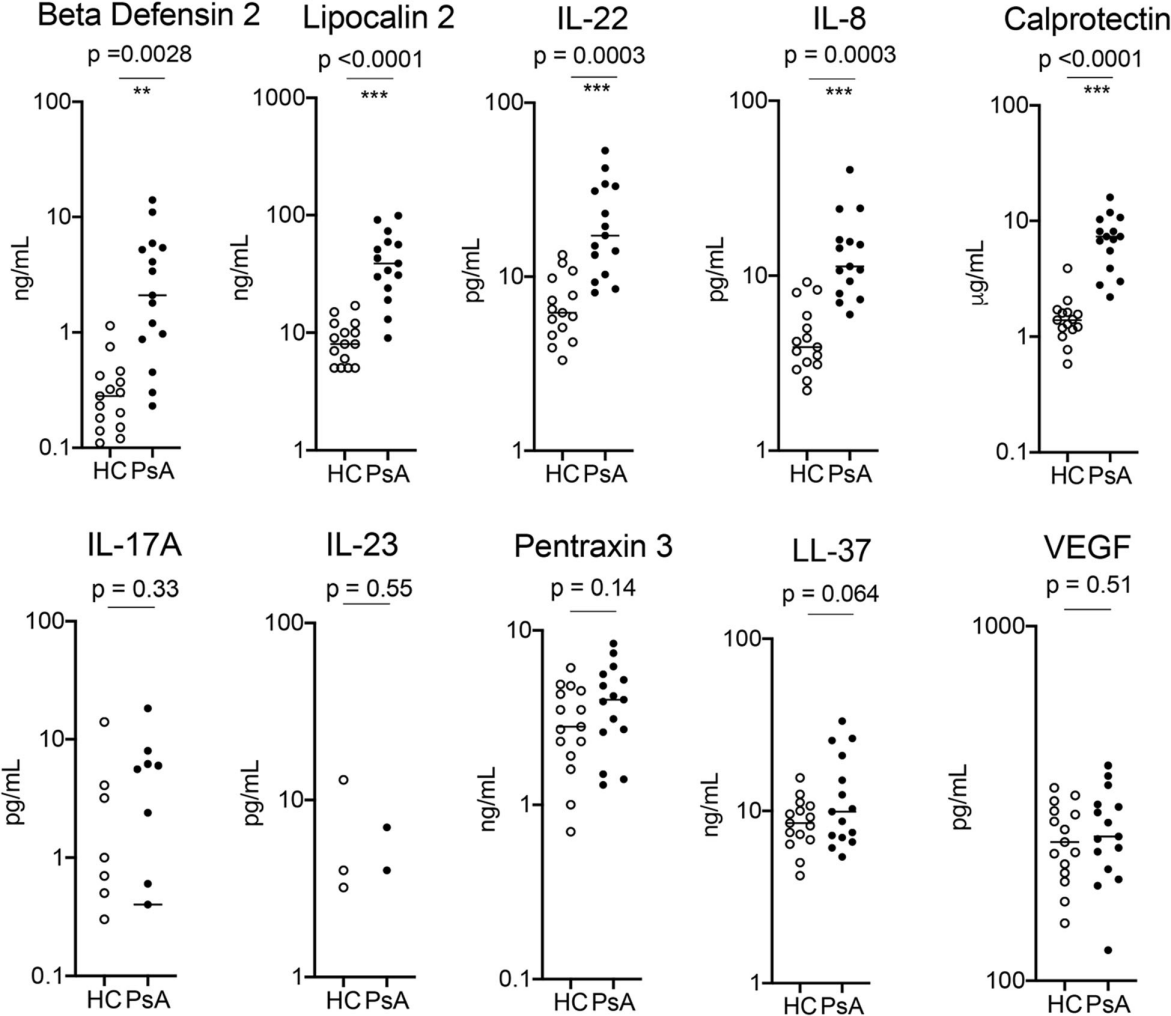
Identification of inflammation markers elevated in psoriatic arthritis (step 1): serum levels of the respective markers were measured in 10 healthy controls (HC) and 10 patients with psoriatic arthritis (PsA). The respective markers were lipocalin 2, beta-defensin 2, calprotectin, pentraxin 3, LL-37 (cathelicidin), vascular endothelial growth factors (VEGF) as well as interleukins (IL)-8, -17A, -22, and -23. Significances between HC and PsA are indicated and calculated by unpaired Student’s test

In the first step, 10 healthy controls and 10 patients with PsA (skin and joint involvement) were randomly selected and tested for 10 different serum parameters associated with (i) IL-17/IL-23 activation (lipocalin 2, beta-defensin2, IL-17A, IL-22, and IL-23), (ii) innate immune cell activation (calprotectin, pentraxin 3, LL-37/cathelicidin, IL-8), and (iii) angiogenesis (VEGF). Among them, 5 markers (lipocalin 2, beta-defensin2, IL-22, calprotectin, and IL-8) were significantly elevated in PsA patients compared to controls, while the others were either not significantly different (pentraxin 3, LL-37/cathelicidin, VEGF) or not detectable in a substantial proportion of controls or patients (IL-17A, IL-23) (Fig. 1). Based on these data, we pursued 5 markers (lipocalin 2, beta-defensin 2, IL-22, calprotectin, and IL-8) in addition to CRP assessment in a larger patient cohort with different patterns of psoriatic disease.

### Serum markers indicating different patterns of psoriatic disease

**Fig. 2:**
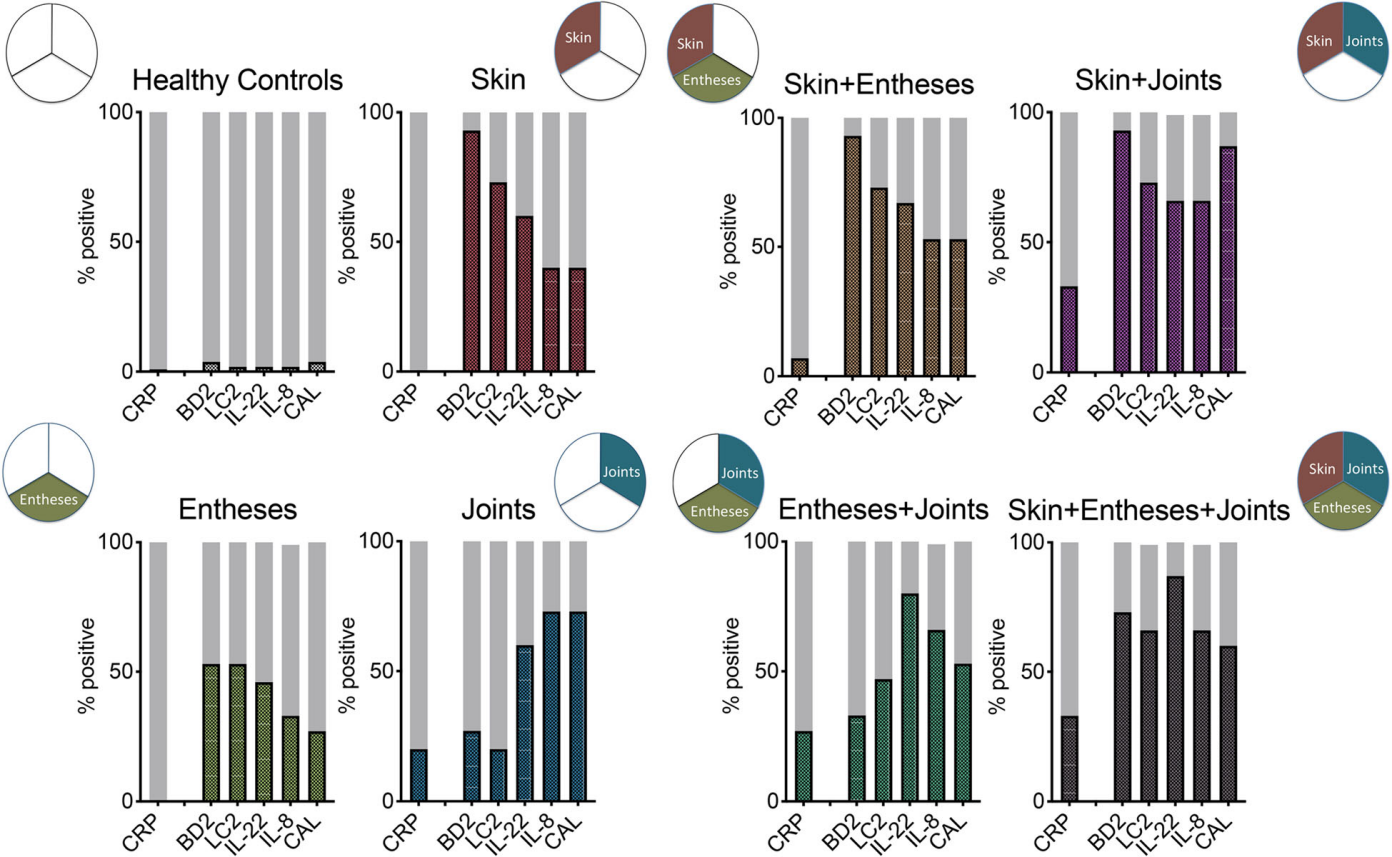
Inflammation markers in various subsets of patients with the psoriatic disease (step 2). Significantly different markers from step 1 (beta-defensin 2, BD2; lipocalin 2, LC2; interleukin-22, IL-22; interleukin-8, IL-8; calprotectin, CAL) as well as C-reactive protein (CRP) were analyzed in 105 healthy controls and each 15 patients with either monomorphic psoriatic disease of the skin (red), the entheses (green), or the joint (blue) or polymorphic disease of with skin/entheses, skin/joints, entheses/joints, or all 3 manifestations. Y-axis indicates % of positive (> 3 SD over mean; > 0.5 mg/dl in case of CRP) patients for the respective markers

We next analyzed 105 controls and 105 patients with the psoriatic disease (each 15 in the 7 aforementioned disease patterns) for systemic levels of lipocalin 2, beta-defensin 2, IL-22, calprotectin, and IL-8 as well as CRP. As expected, CRP levels were normal in the majority of individuals. The respective percentages of patients with elevated CRP (> 5 mg/L) were as follows: S 0%, E 0%, A 20%, SE 7%, SA 33%, EA 27%, and SEA 33%, indicating that only a subset of patients with arthritis, but not patients with skin or entheseal disease show elevated CRP (Fig. 2). Also, CRP level was only correlated to the extent of arthritis but not skin disease or enthesitis (Additional file 3: Table S1). In sharp contrast, beta-defensin 2 levels (> 1.88 ng/mL) and lipocalin 2 (> 24.7 ng/mL) were elevated in the majority of patients with monomorphic skin (93% and 73%, respectively) and entheseal (both 53%), but not joint disease (27% and 20%, respectively). Serum levels of both proteins were significantly correlated to the extent of skin disease and to a lesser extent also entheseal disease (Additional file 3: Table S1). Conversely, elevations of calprotectin (> 3.58 μg/mL) and IL-8 (> 10.3 pg/mL) were found in the majority of patients with joint disease (73%) and were correlated to the extent of arthritis (Additional file 3: Table S1). IL-22 was elevated (> 17.1 pg/mL) in all three manifestations of psoriatic disease. Reflecting a combination of the findings the vast majority of patients with polymorphic disease manifestation (SE, SA, EA, SEA) showed widespread marker elevation. The concentration of biomarkers in all the subsets of patients is shown in Tables 1 and 2. Results for individual patients in all 5 markers plus CRP are shown in Additional file 1: Figure S1. Specificities, sensitivities, and predictive values for the individual parameters are shown in Additional file 4: Table S2.

### Effects of cytokine blockers on systemic markers of inflammation

**Fig. 3:**
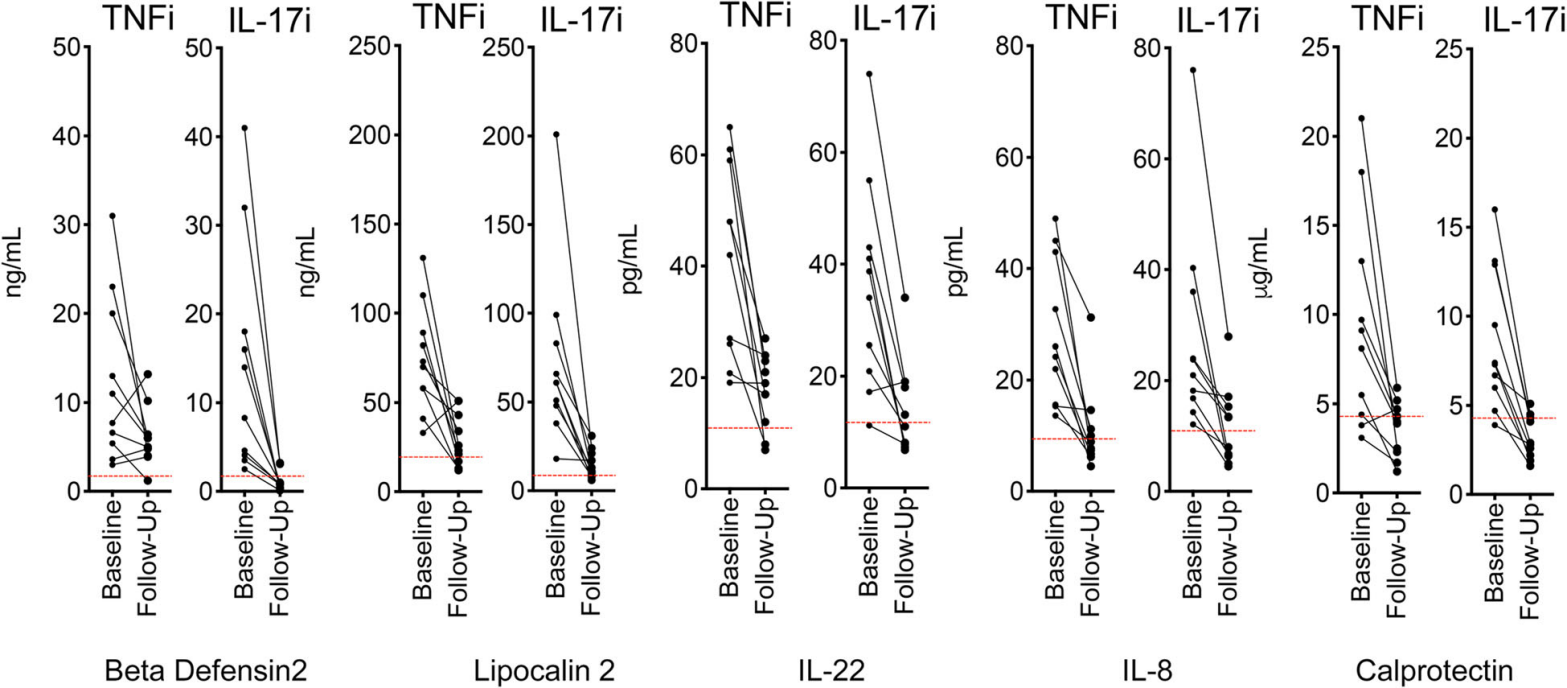
Effect of anti-cytokine treatment on inflammation markers (step 3). Patients with psoriatic arthritis received treatment with either tumor necrosis factor inhibitor adalimumab (TNFi; *N* = 10) or interleukin-17 inhibitor secukinumab (IL-17i; *N* = 10). Serum was analyzed for beta-defensin 2 (BD2), lipocalin 2 (LC2), interleukin (IL)-22, interleukin (IL)-8, and calprotectin (CAL) at baseline and 3 months follow-up

Finally, we assessed how the treatment of PsA with IL-17 or TNF inhibition affects elevated markers of inflammation. To do this, we compared baseline and 6-month follow-up serum samples in PsA patients (with skin and joint involvement) starting on either IL-17- (*N* = 10, secukinumab) or TNF- (*N* = 10, adalimumab) inhibition. Patients in both groups were balanced for skin (PASI 9.6 ± 1.2 vs. 8.9 ± 0.6; *p* = 0.64), joint (SJC 5.5 ± 0.8 vs. 6.8 ± 1.0; *p* = 0.34), and entheseal (SPARCC 1.9 ± 0.7 vs. 1.5 ± 0.5; *p* = 0.67) manifestations. Both treatment regimens significantly lowered the elevated levels of lipocalin 2, beta-defensin2, IL-22, calprotectin, and IL-8 (Fig. 3 and Additional file 5: Table S3). While the effects on IL-22, calprotectin, and IL-8 were similar, IL-17 inhibition showed a more pronounced lowering of lipocalin 2 and beta-defensin 2 levels.

## Discussion

These data show the feasibility of measurement of systemic inflammation in patients with psoriatic disease, if not focusing the analysis on the acute phase response. The results also suggest that various subsets of psoriatic disease show distinct marker profiles with differences for skin, entheseal, and joint disease.

This study particularly underlines that CRP is not a perfectly useful marker for assessing systemic inflammation in psoriatic disease. Skin and entheseal disease are not associated with CRP elevations despite active inflammation. If present, CRP elevations (> 5 mg/L) are confined to synovial disease (joint swelling). However, only a fraction of about one-third of patients with arthritis shows elevated CRP. As mentioned, these findings reflect the generally low or absent IL-6 signature in psoriatic disease, which is required for mounting the production of acute-phase proteins in hepatocytes.

In contrast, beta-defensin 2 and lipocalin 2 are both IL-17 regulated mediators, which are produced by innate immune cells. Both are found at much higher levels than IL-17 in the circulation and appear to be strongly associated with skin and to some extent also with entheseal disease, while the joint disease is more inconsistently associated with elevated levels (< 30%) (Additional file 2: Figure S2). These results confirm earlier data showing elevated beta-defensin levels in psoriasis patients and its association with the extent of skin involvement [12]. Hence, beta-defensin and lipocalin 2 provide an opportunity to quantify systemic inflammation elicited by psoriatic skin and entheseal disease.

In contrast, the elevation of the alarmin calprotectin and the neutrophil chemo-attractant mediator IL-8 in the serum were preferentially driven by psoriatic joint disease and to some extent also entheseal disease rather than skin disease. These findings support the role of neutrophil influx and activation in the musculoskeletal manifestations of psoriatic disease and provide instruments to measure this process, which occurs independently from acute phase reactants. While IL-17A and IL-23 were barely detectable in the circulation, we found IL-22 in relevant concentrations across all manifestations of psoriatic disease.

Overall, these results offer a new possibility to measure systemic inflammation in psoriatic disease complementing previous findings of altered bone biomarkers in psoriatic arthritis [26, 27]. As such, these data address the unmet need of immunological markers to monitor disease activity in psoriatic disease. Such an approach can complement the use of well-established clinical instruments to quantify the extent of skin, entheseal, and joint disease. In consequence, these markers may not only support the classification of different endotypes in psoriatic disease but may also improve the characterization of the activity of the disease as well as its response to treatment. Finally, these findings will also allow addressing new research questions, such as the characterization of axial disease (which has not been included into this study), the relation of these markers to structural damage, as well as their potential role in defining patients that progress from psoriatic skin to musculoskeletal disease.

## Conclusion

These data indicate that a distinct set of markers allows measuring systemic inflammation in patients with psoriatic disease in the absence of elevated C-reactive protein. Furthermore, different clinical subsets of psoriatic disease based on skin, entheseal and joint involvement are characterized by specific inflammation marker profiles. Treatment of psoriatic disease with cytokine inhibitors reduces these elevated levels of systemic inflammation markers.

## Supplementary information

**Additional file 1:** **Figure S1.** Individual levels of inflammation markers in patients with different subsets of psoriatic disease.

**Additional file 2:** **Figure S2.** Marker Spidergram.

**Additional file 3:** **Table S1**. Correlation between serum markers and disease activity parameters.

**Additional file 4:** **Table S2.** Sensivity and specificity of the serum markers.

**Additional file 5:** **Table S3.** Effects of TNF- and IL-17A inhibition on the serum levels of the markers.

## Data Availability

The datasets used and/or analyzed during the current study are available from the corresponding author on reasonable request.

## Abbreviations

A: Arthritis
BD: Beta defensin
CP: Calprotectin
CRP: C-reactive protein
DAPSA: Disease activity in psoriatic arthritis
E: Enthesitis
IL: Interleukin
LC: Lipocalin
PASI: Psoriasis area severity index
PSA: Psoriatic arthritis
S: Skin disease
SJC: Swollen joint count
SPARCC: Spondyloarthritis Research Consortium of Canada
VEGF: Vascular endothelial growth factor
