# Supplementary figures and images for: A set of serum markers detecting systemic inflammation in psoriatic skin, entheseal, and joint disease in the absence of C-reactive protein and its link to clinical disease manifestations
Source: Arthritis Res Ther. 2020 Feb 12;22:26. doi: 10.1186/s13075-020-2111-8 (PMC7017480; doi:10.1186/s13075-020-2111-8)

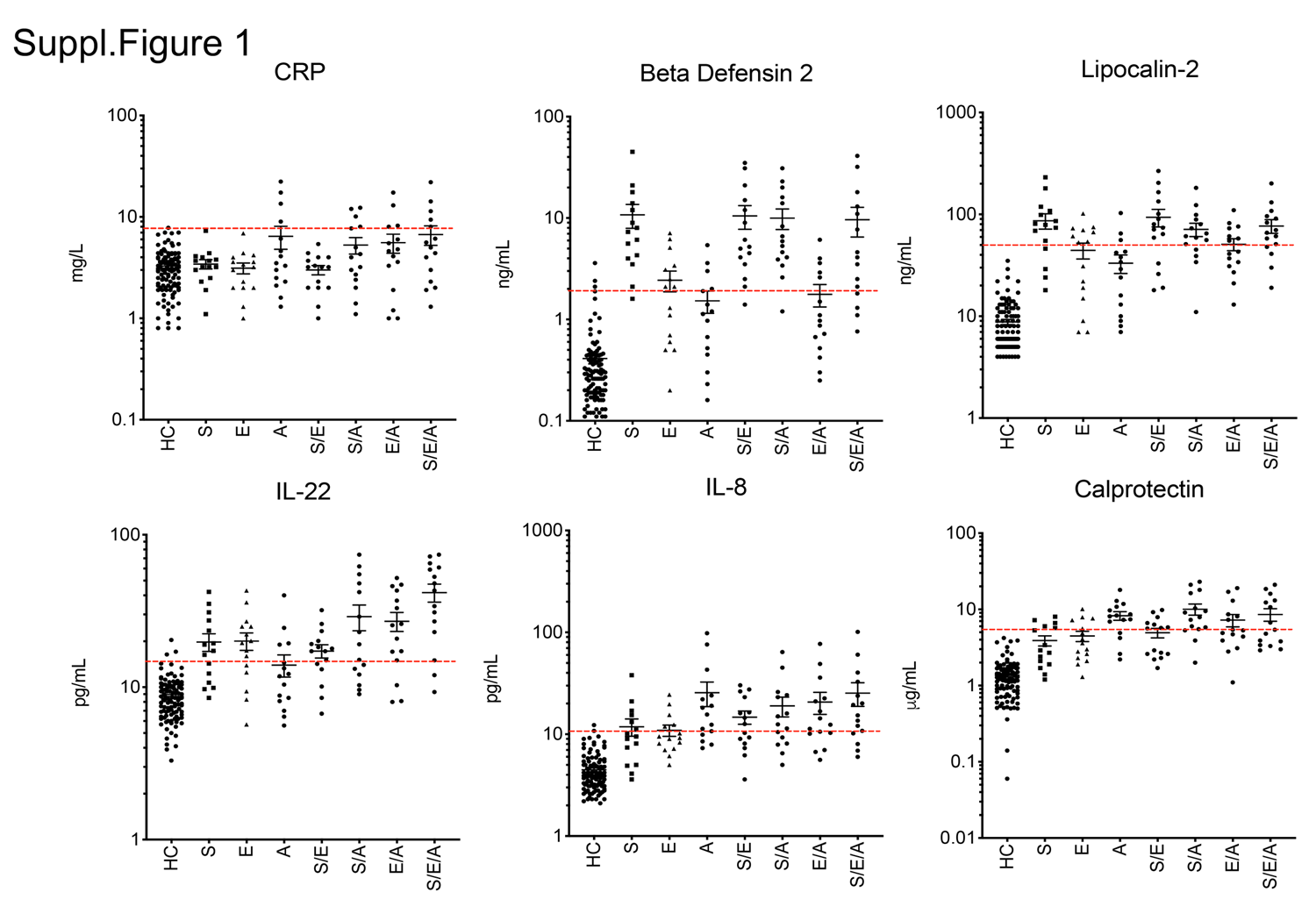

Supplement: Supplementary file 1 — Additional file 1: Figure S1. Individual levels of inflammation markers in patients with different subsets of psoriatic disease. [file 13075_2020_2111_MOESM1_ESM.tif]

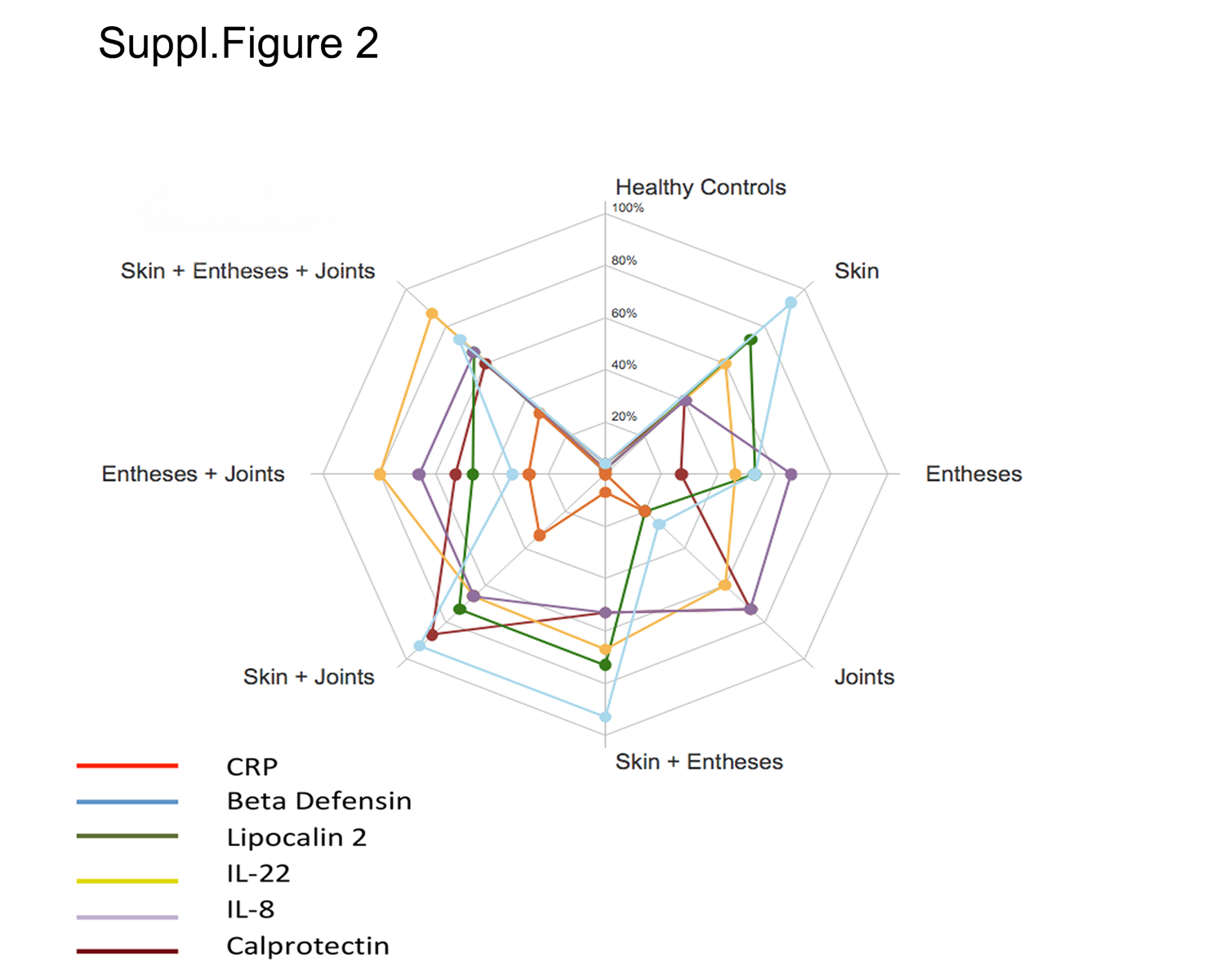

Supplement: Supplementary file 2 — Additional file 2: Figure S2. Marker Spidergram. [file 13075_2020_2111_MOESM2_ESM.tif]
