# Supplementary material for: A set of serum markers detecting systemic inflammation in psoriatic skin, entheseal, and joint disease in the absence of C-reactive protein and its link to clinical disease manifestations
Source: Arthritis Res Ther. 2020 Feb 12;22:26. doi: 10.1186/s13075-020-2111-8 (PMC7017480; doi:10.1186/s13075-020-2111-8)
Supplement: Supplementary file 3 — Additional file 3: Table S1. Correlation between serum markers and disease activity parameters. [file 13075_2020_2111_MOESM3_ESM.docx]

Supplementary Table 1

|  | **PASI** | **SPARCC** | **SJC66** | **DAPSA** |
| --- | --- | --- | --- | --- |
|  | N=60 | N=60 | N=60 | N=60 |
| **CRP** | r=0.01; p=0.90 | r=0.31; p=0.01* | r=0.47; p=0.0001*** | r=0.42; p=0.0006*** |
| **LC2** | r=0.76; p<0.0001*** | r=0.30; p=0.01* | r=0.21; p=0.09 | r=0.14; p=0.25 |
| **BD2** | r=0.76; p<0.0001*** | r=0.01; p=0.90 | r=0.01; p=0.90 | r=0.20; p=0.11 |
| **IL-22** | r=0.39; p=0.018* | r=0.30; p=0.01* | r=0.28; p=0.02* | r=0.19; p=0.14 |
| **IL-8** | r=0.04; p=0.72 | r=0.21; p=0.10 | r=0.44; p=0.0003*** | r=0.35; p=0.005** |
| **CP** | r=0.03; p=0.82 | r=0.18; p=0.16 | r=0.38; p=0.0028** | r=0.33; p=0.008** |

PASI: psoriasis area severity index; SPARCC: Spondyloarthritis Research Consortium of Canada; SJC66: swollen joint count; DAPSA: disease activity in psoriatic arthritis; CRP: C-reactive protein, LC2: lipocalin 2, BD2: beta-defensin 2, IL: interleukin, CP: calprotectin and IL-8. All values indicate means ± SEM. r: Spearmans rho; Asterisks indicate significant correlations. Numbers comprise all patients with skin disease in case of PASI, all patients with enthesitis in case of SPARCC and all patients with arthritis in case of SJC.
