# Supplementary material for: A set of serum markers detecting systemic inflammation in psoriatic skin, entheseal, and joint disease in the absence of C-reactive protein and its link to clinical disease manifestations
Source: Arthritis Res Ther. 2020 Feb 12;22:26. doi: 10.1186/s13075-020-2111-8 (PMC7017480; doi:10.1186/s13075-020-2111-8)
Supplement: Supplementary file 4 — Additional file 4: Table S2. Sensivity and specificity of the serum markers. [file 13075_2020_2111_MOESM4_ESM.docx]

Supplementary Table 2

|  | **Sensitivity** | **Specificity** | **PPV** | **NPV** |
| --- | --- | --- | --- | --- |
| **CRP** | 26% | 89% | 71% | 55% |
| **LC2** | 80% | 98% | 97% | 83% |
| **BD2** | 67% | 96% | 94% | 75% |
| **IL-22** | 54% | 98% | 96% | 68% |
| **IL-8** | 58% | 98% | 96% | 70% |
| **CP** | 68% | 96% | 94% | 75% |

CRP: C-reactive protein, LC2: lipocalin 2, BD2: beta-defensin 2, IL: interleukin, CP: calprotectin and IL-8. PPV: positive predictive value, NPV: negative predictive value.
