# Supplementary material for: A set of serum markers detecting systemic inflammation in psoriatic skin, entheseal, and joint disease in the absence of C-reactive protein and its link to clinical disease manifestations
Source: Arthritis Res Ther. 2020 Feb 12;22:26. doi: 10.1186/s13075-020-2111-8 (PMC7017480; doi:10.1186/s13075-020-2111-8)
Supplement: Supplementary file 5 — Additional file 5: Table S3. Effects of TNF- and IL-17A inhibition on the serum levels of the markers. [file 13075_2020_2111_MOESM5_ESM.docx]

Supplementary Table 3

|  | **TNFi Baseline** | **TNFi Follow -up** | **IL-17i Baseline** | **IL-17i Follow-up** |
| --- | --- | --- | --- | --- |
|  | N=10 | N=10 | N=10 | N=10 |
| **CRP** | 6.2±1.1 | 3.0±0.2^***^ | 6.6±0.9 | 3.2±0.1^***^ |
| **LC2** | 74±9.5 | 29±4.6^***^ | 72±15 | 14±2.5^***^ |
| **BD2** | 12±2.9 | 6.0±1.0^***^ | 14±4.1 | 1.5±1.4^***^ |
| **IL-22** | 41±5.4 | 17.5±2.1^***^ | 36±5.9 | 14±2.6^***^ |
| **IL-8** | 28±4.1 | 10±2.4^***^ | 28±6.0 | 11±2.2^***^ |
| **CP** | 9.5±1.9 | 3.5±0.4^***^ | 8.7±1.2 | 3.2±0.3^***^ |

TNF, tumor necrosis factor alpha inhibition; IL-17i, interleukin-17 inhibition; CRP: C-reactive protein, LC2: lipocalin 2, BD2: beta-defensin 2, IL: interleukin, CP: calprotectin and IL-8. All values indicate means ± SEM. Asterisks indicate significances (p<0.01) compared to baseline.
